# Urban heat island intensity maps and local weather types description for a 45 French urban agglomerations dataset obtained from atmopsheric numerical simulations

**DOI:** 10.1016/j.dib.2023.109437

**Published:** 2023-07-20

**Authors:** Melis Suher-Carthy, Thomas Lagelouze, Julia Hidalgo, Robert Schoetter, Najla Touati, Renaud Jougla, Valery Masson

**Affiliations:** a Laboratoire Interdisciplinaire Solidarités Sociétés Territoires (LISST), Université de Toulouse, CNRS, 5 Allée Antonio Machado, 31058, Toulouse, France; b Centre National de Recherches Météorologiques (CNRM), Météo-France, CNRS, 42, Avenue Gaspard Coriolis, 31057 Toulouse Cedex 1, France

**Keywords:** Urban microclimate, Air temperature, Town energy balance, SURFEX, Meso-NH, Urban unit

## Abstract

This article presents a dataset of spatial nocturnal Urban Heat Island (UHI) intensities for 45 French urban agglomerations, at a horizontal resolution of 250 m. The urban influence on air temperature at 2 m above ground level was obtained by coupling the mesoscale atmospheric model Meso-NH with the land surface model SURFEX-TEB. For each agglomeration, two specific local weather situations that favour the development of a strong UHI in summer are simulated and described in a specfic sheet. Simulation outputs have been postprocessed to 1) identify the time of day when the UHI is the most developed, 2) to merge information from both meteorological situations in order to obtain one synthetic UHI map and 3) a geographical analysis that allows to classify each city among five spatial UHI classes (Concentrated Very High Intensity; Concentrated High Intensity; Limited Intensity; Dispersed High Intensity; and Dispersed Cool Zones). This dataset can therefore be used for several purposes, from the analysis at the scale of a city to the comparison of the urban agglomerations among them.

Specifications Table

**Table:** 

Subject	Urban Heat Island (UHI)
Specific subject area	Maximal extension and intensity of the nocturnal Urban Heat Island obtained from atmospheric modelling at 250 × 250m of horizontal resolution for 45 French urban agglomerations.
Type of data	Shapefiles Writting document
How data were acquired	For each city, short term (6 days) atmospheric simulations for two Local Weather Types (LWT) that favour the UHI development are performed. The three-hour period corresponding to the maximum UHI intensity is identified and the arithmetic average over this period is calculated. The previous arithmetic average for the two LWT are combined to obtain the maximal UHI extent and intensity. Geographical analysis on UHI patterns was applied to identify spatial UHI classes.
Data format	Analyzed
Description of data collection	The UHI raw data was generated using the non-hydrostatic mesoscale atmospheric model Meso-NH-SURFEX and is described in Gardes et al. 2020 [1]. The method to identify the LWT is described in Jougla et al. 2019 [2]. The LWT identification is integrated in the enriched metadata of each shapefile and the LWT description is presented in a separate .pdf document for each urban area. The geographical patch analysis is described in Suher-Carthy, 2021 [3].
Data source location	Laboratoire Interdisciplinaire Solidarités Sociétés Territoires (LISST), Université de Toulouse, CNRS, 5 Allée Antonio Machado, 31058 Toulouse, France.
Data accessibility	https://doi.org/10.5281/zenodo.8009879

## 1 Value of the Data

• The dataset contains the nocturnal Urban Heat Island intensity in 45 urban agglomerations in metropolitan France and the description of the most current Local Weather Types.
• This collection can be used to investigate the meteorological context and the microclimate of a city at a horizontal resolution of 250 m.
• The gathered data can be integrated and/or combined with other climate, geographical, or social data.
• The data provided can be used in cross scientific, technical or operational studies that aim to assess the social or environmental consequences of the UHI.

## 2 Objective

The data was generated within the framework of two French research projects. These are the ANR-MApUCE “Applied Modelling and Planning Laws: Climate and Energy" and the ADEME-PAENDORA “Planning, Adaptation and Energy: Territorial data and support” projects. The general objective of the projects was to integrate quantitative urban microclimate, climate, and energy data into the most relevant urban policies and legal French documents^1^. Within this framework, an urban (MApUCE database), an architectural (Danube database), and a meteorological database (Local Weather Type classification) were developed to perform the mesoscale climatic simulations here presented.

## 3 Data Description

This data paper provides maps of UHI intensity at a resolution of 250 m, for 45 French urban agglomerations (Fr: “Unité Urbaine”). In the French context, urban agglomerations are defined as “a municipality or a set of municipalities with a continuous built-up area (no distance of more than 200 meters between two buildings) and at least 2000 inhabitants” [4].

In this data collection, each urban agglomeration has its own shapefile layer, whose attributes are organized as displayed in Table 1. The urban agglomerations are named after the most important city within them Table 2.

**Table 1:** Attribute structure of the provided Shapefile layer.

Variable	Column label [unit]
Identifier	ID [.]
Urban Heat Island Intensity	UHI [ΔT_air_°C]

**Table 2:** Table of urban agglomerations names.

ACRONYM	URBAN AGGLOMERATION
Amiens (Ami)	Amiens
Angers (Ang)	Angers
Arras (Arr)	Arras
Avignon (Avi)	Avignon
Bayonne (Bay)	Bayonne
Beauvais (Bea)	Beauvais
Belfort (Bel)	Belfort
Besançon (Bes)	Besançon
Bethune (Bet)	Béthune
Bordeaux (Bor)	Bordeaux
Boulogne_sur_Mer (Bou)	Boulogne-sur-Mer
Caen (Cae)	Caen
Calais (Cal)	Calais
Chalon_sur_Saone (Cha)	Chalon-sur-Saône
Clermont-Ferrand (ClF)	Clermont-Ferrand
Colmar (Col)	Colmar
Compiegne (Com)	Compiègne
Creil (Cre)	Creil
Dijon (Dij)	Dijon
Douai_Lens (Dou)	Douai-Lens
Dunkerque (Dun)	Dunkerque
La_Rochelle (LaR)	La Rochelle
Le_Havre (LeH)	Le Havre
Lille (Lil)	Lille
Lorient (Lor)	Lorient
Lyon (Lyo)	Lyon
Metz (Mtz)	Metz
Montbeliard (Mon)	Montbéliard
Montpellier (Mtp)	Montpellier
Mulhouse (Mul)	Mulhouse
Nancy (Ncy)	Nancy
Nantes (Nan)	Nantes
Nice (Nic)	Nice
Nimes (Nim)	Nîmes
Orleans(Orl)	Orléans
Paris (Par)	Paris
Pau (Pau)	Pau
Reims (Rms)	Reims
Rouen (Rou)	Rouen
Saint_Etienne (SEt)	Saint-Etienne
Saint_Nazaire (SNz)	Saint-Nazaire
Thionville (Thi)	Thionville
Toulon (Tou)	Toulon
Tours (Trs)	Tours
Valenciennes (Val)	Valenciennes

## 4 Experimental Design, Materials and Methods

**Fig. 1:**
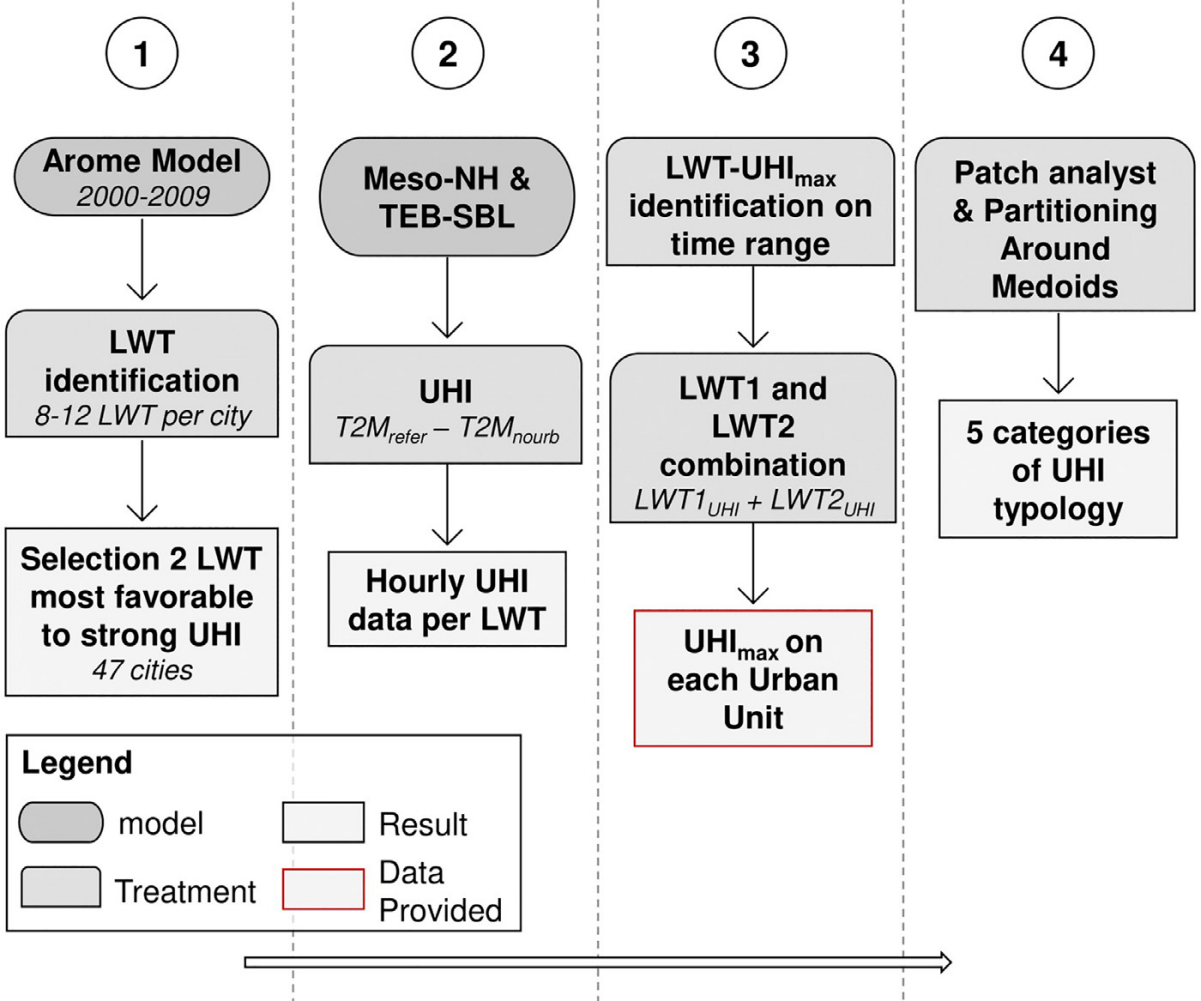
The methodology employed in the French ANR-MAPuCE project to produce data on nocturnal UHI intensity in French cities.

Four main steps were necessary to generate the dataset. The first step is the identification and description of two Local Weather Types (LWT) that favour the UHI formation. The second step is to simulate six days during the summer season for each LWT using the mesoscale atmospheric model Meso-NH coupled to the land Surface Externalisée, in French, and Town Energy Balance (SURFEX-TEB) surface models. The third step is the identification of the three-hour period during the night with the highest UHI intensity and the averaging of this period, and the fourth step is the cluster classification of UHI spatial pattern (Fig. 1).

### 4.1 First Step: Local Weather Types Favourable to a Strong Urban Heat Island Effect

The UHI intensity depends on the local meteorological conditions. In order to identify the local meteorological situations favourable to a strong UHI, for each urban unit, LWT are identified and described according to the method of Hidalgo et al. (2014) [5] and Hidalgo and Jougla (2018) [6]. The selected LWT are based on the PAM classification (Partitioning Around Medoids) that considers daily values of temperature, humidity, precipitation, and wind direction. This method was applied by Jougla et al. (2019) [2] to 50 French cities using a re-analysis conducted with the operational high-resolution (0.025° horizontal resolution, that is about 2.5 km in France metropolitan) AROME model for the period 2000 to 2009. The UHI data provided in this article is obtained from the combination of numerical simulations for six days and two LWT the most favourable for the development of a strong UHI during the summer season [1].

### 4.2 Second Step: Numerical Simulations of the Nocturnal Urban Heat Island Intensity

For each selected LWT, numerical simulations are performed with the mesoscale atmospheric model Meso-NH [7]. It uses four horizontal domains with horizontal grid resolution of 8 km (D1), 2 km (D2), 1 km (D3), 250 m (D4). Their size and position are adapted to the urban agglomeration's specific spatial characteristics [1].

Meso-NH is coupled with the urban canopy parameterization Town Energy Balance (TEB) [8] which simulates the urban surface energy balance according to the meteorological conditions simulated by Meso-NH. Vertical profiles of meteorological parameters in the urban canopy layer are calculated with the Surface Boundary Layer (SBL) parameterization [9]. The second level of the TEB-SBL is located in 2 m a.g.l.; the simulated air temperature from this level is used as urban air temperature (T2M_URB_). Simulations are repeated by replacing the urban land cover with a rural land cover type that is characteristic of the city surroundings. An SBL scheme similar to the one of TEB is employed in the rural areas; the non-urban air temperature (T2M_NOURB_) is derived from this simulation. The difference between the urban and non-urban simulation yields the UHI intensity. For the two selected LWT, the UHI of the day following the 6 LWT days is analysed since UHI depends on the weather conditions of the previous day [10].

### 4.3 Third Step: Maximum Urban Heat Island Intensity

The raw data from the numerical simulations are in the form of a data frame containing grid point values for the 24 hours of the day. The three-hour periods with the maximum UHI intensity during both LWT are first identified. Secondly, the data is rasterized and the average over those 3 hours is calculated for each LWT. The final raster is obtained combining both rasters (maximum value per pixel) to reflect the one with highest exposure to UHI intensity [3].

### 4.4 Fourth Step: UHI Cluster Typologies

**Fig. 2:**
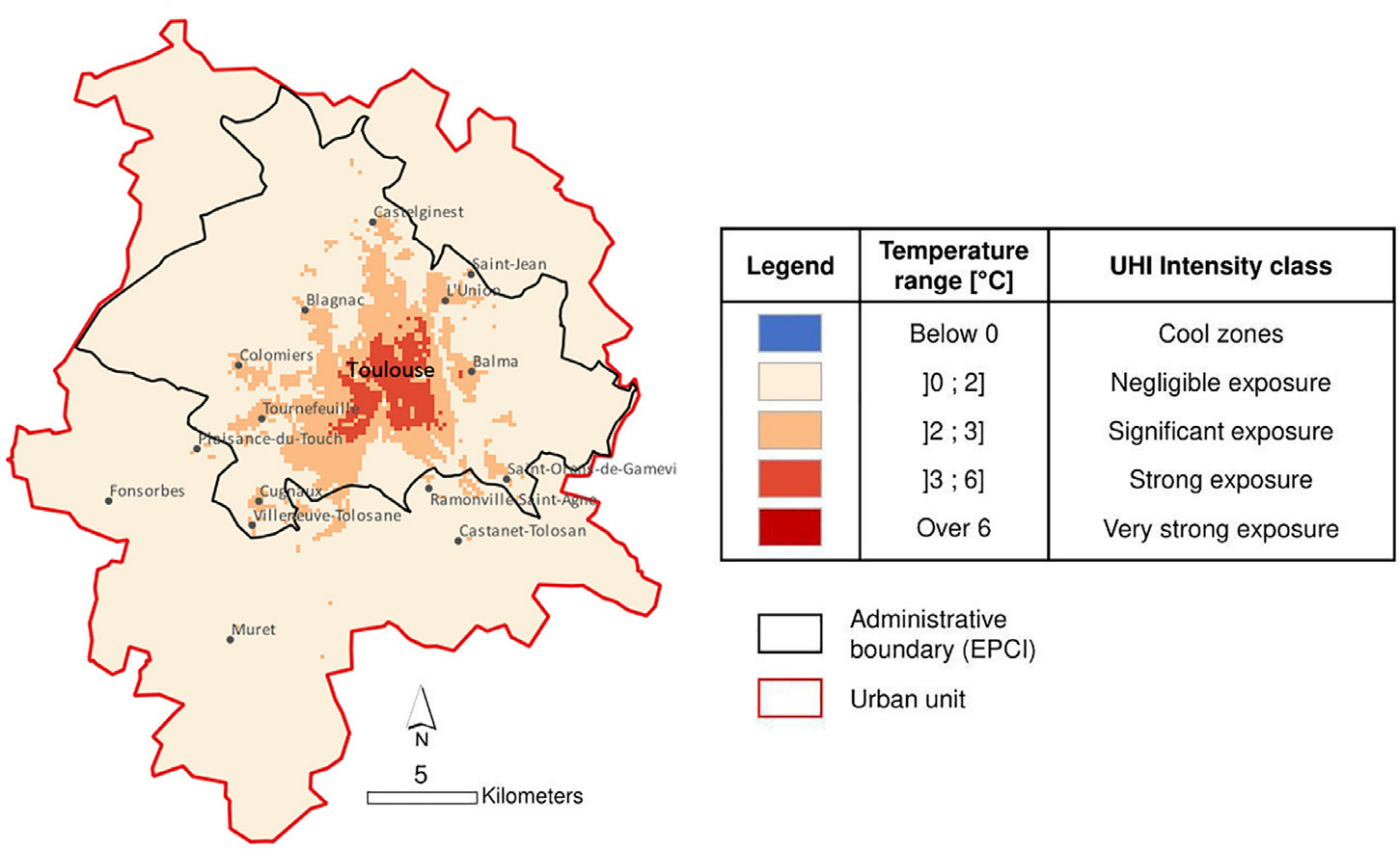
An example of a UHI map of the urban agglomeration of Toulouse.

The maximum UHI for the urban agglomerations is analyzed according to their intensity and spatial distribution – to identify common UHI spatial patterns that would allow them to be grouped into clusters. For this objective, the ArcGIS PRO Patch Analyst^2^ tool to identify shared typologies between cities, and cluster categorization was executed in Rstudio with the {factoextra} package and Partitioning Around Medoids method. At the end of the treatments, 5 categories of UHI typology were found: (**1**) Concentrated High Intensity, (**2**) Limited Intensity, (**3**) Dispersed High Intensity, (**4**) Dispersed Cool Zones, (**5**) Concentrated Very High Intensity, cluster specific to the Urban Unit of Paris [3]. The types of UHI intensity constituting these clusters can be understood and described through the UHI classes provided in Fig. 2 of the next section.

1. The cluster 1 concerns strong exposure concentrated at the urban cores generally. Often, strong exposure zones in highly compact forms are crossed by rivers, passing through the cores of the cities.
2. The cluster 2 illustrates significant exposure with limited expansion and is sometimes dispersed in the urban space.
3. The cluster 3 includes strong exposure highly fragmented and dispersed in the place. In this cluster, strong UHI exposure class is dominant.
4. The cluster 4 is marked by the presence of cool zone area that causes heterogeneous forms of UHI. All the urban agglomerations of this cluster are coastal and are located in southern France.
5. The cluster 5 is only connected to the agglomeration of Paris. This city is a cluster in itself because of these very strong UHI exposure. The large urban structures in this city make it impossible to compare it to other urban areas in France.

### 4.5 Methodological Summary

The methodology for obtaining the data previously described in 3.1 to 3.3 is summarized in Fig. 1. Fig. 2 gives an example of a cartographic representation of the data provided, for the urban unit of Toulouse; and proposes a discretization of the UHI intensities. Information on the UHI cluster to which the urban areas belong, and the LWTs required for the simulations, are available in the metadata of each .shp file.

## Ethics Statement

The authors have read and follow the ethical requirements for publication in Data in Brief. The work does not involve studies with animals and humans.

## CRediT Author Statement

**Thomas Lagelouze:** Data Curation; Writing – Original Draft; **Melis Suher-Carthy:** Data Curation, Investigation, Methodology of steps 3 and 4 in Fig. 1; **Julia Hidalgo:** Conceptualization of steps 1, 3 and 4 in Fig. 1, Supervision, Writing – review & editing; **Robert Schoetter:** Conceptualization and investigation of step 2 in Fig. 1; **Najla Touati:** Visualization; **Renaud Jougla:** Conceptualization and investigation of step 1 in Fig. 1; **Valery Masson:** Supervision of step 2. Project administration.

## Declaration of Competing Interest

The authors declare that they have no known competing financial interests or personal relationships that could have appeared to influence the work reported in this paper.

## Data Availability

MApUCE_UHI_LWT_dataset (Original data) (Zenodo).

## Footnotes

1 http://www.umr-cnrm.fr/ville.climat/spip.php?rubrique120&lang=en;http://www.umr-cnrm.fr/spip.php?article1119&lang=en

2 In this data paper, Patch Analyst Method was used. However, we could have used other methods, such as Fragstat.
